# Chinese pharmacy students' knowledge, attitudes and practice about environmental risks of pharmaceuticals and their source control from the perspective of One Health

**DOI:** 10.3389/fpubh.2026.1803589

**Published:** 2026-04-07

**Authors:** Sijia Ma, Yanru Wang, Jun Wang

**Affiliations:** Department of Pharmacy, School of Medicine, Wuhan University of Science and Technology, Wuhan, China

**Keywords:** environmental risks, One Health, pharmaceuticals as emerging contaminants, pharmacy students, source control

## Abstract

**Background:**

Growing concern has raised regarding environmental risks posed by pharmaceuticals, as representative emerging contaminants originating from human and veterinary pharmacotherapies. Given irreplaceable role of source control in the management of emerging contaminants, the active engagement of pharmacy professionals is fundamental to solving environmental issues associated with pharmaceuticals. This study explored the knowledge, attitudes and practice of pharmacy students, future pharmacy professionals, toward environmental risks of pharmaceuticals and their source control from a One Health perspective.

**Methods:**

A cross-sectional survey was conducted using an online questionnaire, which was self-developed to assess the knowledge levels, current concerns, practices, and attitudes of undergraduate and graduate pharmacy students at a Chinese university regarding One Health, environmental risks of pharmaceuticals and their source control.

**Results:**

A total of 206 valid responses were obtained, with a response rate of 71.8%. Average self-perceived knowledge scores of students on One Health, environmental risks of pharmaceuticals, and their source control ranged from 2.24 to 2.48 on a five-point scale, and actual knowledge score measured via objective assessment was 2.48 out of 10. Only 46.1% respondents reported considering environmental factors during pharmaceutical use, and 18.0% opted to return unused medications to pharmacies or hospitals. Students who integrated environmental considerations into their pharmaceutical use or routinely returned unused drugs to pharmacies/hospitals achieved significantly higher knowledge scores than their counterparts. Average attitude score reached 4.05 on a five-point Likert scale. Pearson's correlation analysis revealed positive correlations between attitude scores and self-perceived knowledge regarding the environmental risks of pharmaceuticals and their source control from the perspective of One Health.

**Conclusion:**

Chinese pharmacy students hold positive attitudes toward the environmental risks of pharmaceuticals and their source control from a One Health perspective, but lack relevant knowledge and practical experience, thus highlighting the need to integrate related sustainability content into pharmacy curricula to promote sustainable pharmaceutical and healthcare practices.

## 1 Introduction

With the rapid advancement of the Industrial Revolution, a multitude of novel chemicals have been introduced into the environment following their utilization, and persist in environmental matrices as emerging contaminants (ECs). Unlike conventional contaminants such as heavy metals, nutrients, and dyes, ECs pose more prominent risks to ecosystems and human health due to their complex toxic effects and general lack of regulatory oversight (1, 2). Yet, ECs only began to attract significant attention in the latter half of the 20th century (3, 4). To date, both the general public and professionals still have a limited understanding of ECs, thus underscoring the urgency of enhanced educational efforts to build support for mitigating the environmental and health impacts posed by this group of contaminants (3, 4).

The expansion of the pharmaceutical market has led to a growing occurrence of pharmaceuticals as ECs (PECs) in the environment, resulting from the production, use, and waste generation of pharmaceutical products. Owing to their pseudo-persistence, potent biological activity, and extensive application in human and veterinary medicine, pharmaceuticals have emerged as one of the most representative and abundant groups of ECs (5, 6). Compelling evidence has demonstrated that nearly all major categories of active pharmaceutical ingredients (APIs) used in large quantities, such as antibiotics, anti-inflammatory drugs, hormones, central nervous system agents, lipid regulators, and antihypertensives, are widely present in various environmental matrices, including water, soil, sediments, and biota, across the globe (7–9). Although typically detected at trace levels, PECs in the environment exert distinct hazards to ecosystems, non-target aquatic organisms, wildlife, and even humans, because pharmaceuticals are specifically designed to modulate biochemical pathways in humans and animals even at extremely low doses (7–9).

Environmental issues associated with PECs are inherently complex, as they interconnect all three vertices of the “One Health triangle” (humans, animals, and the environment). Therefore, addressing these issues requires interdisciplinary collaboration, cross-sectoral coordination, and transnational cooperation that span drug development, academic research, policy-making, and healthcare delivery (10–14). Recent studies have underscored the importance of adopting a holistic systems perspective grounded in the One Health framework, which highlights the interdependencies between human, animal, and plant health, to support key stakeholders in collectively discussing, researching, prioritizing, and implementing systemic solutions for pharmaceutical environmental pollution (10, 11, 14). In Scotland, for instance, the One Health Breakthrough Partnership, a multi-agency collaborative initiative involving Highland National Health Service, Scottish Water, the Scottish Environment Protection Agency, and relevant enterprises, research institutions, and universities, has been established as a successful upstream intervention to address water pollution caused by excreted pharmaceuticals (13, 15). Currently, mitigating the environmental impacts of PECs is widely recognized to require great efforts from multi-disciplinary fields (14). Notably, given the irreplaceable role of source control in EC management, the active engagement of pharmacy professionals is considered fundamental to solving the environmental problem associated with pharmaceuticals (11, 14, 16). APIs from medical products can enter air, soil, and water through multiple pathways, including human and animal excretion, improper disposal of unused or expired medications, and discharges from pharmaceutical manufacturing, agriculture, and aquaculture (7, 10, 16, 17). Accordingly, pharmacy professionals, including drug developers, pharmacists, pharmacy technicians, manufacturers, and regulators, should bear professional responsibility for reducing the environmental burden and risks of PECs. This responsibility can be fulfilled through their involvement in implementing a sustainable pharmacy framework that addresses the entire lifecycle of medicines, from production and prescription to distribution, use, and take-back (11). However, to competently assume this role, pharmacy professionals must possess adequate awareness and knowledge of PECs and their source control strategies.

In recent years, the necessity of integrating environmental issues associated with PECs or the One Health perspective into pharmacy education has gained increasing attention, particularly in high-income countries (HICs) (2, 11, 18–21). Such integration can empower future pharmacy practitioners to address complex pharmaceutical-related environmental challenges in their professional roles, including supplying and dispensing medications, providing rational drug use counseling, conducting public health education, and managing pharmaceutical waste (2, 11, 18–21). However, it remains unclear how to effectively implement such integration and what specific content should be incorporated. Moreover, low- and middle-income countries (LMICs) may bear greater responsibilities in mitigating the global source-related pollution footprint of PECs, due to factors such as more severe industrial manufacturing pollution, higher population density, and limited wastewater treatment infrastructure (11, 22). In China, pharmacy professionals primarily acquire core professional knowledge and skills during their university education, as the formative stage of their careers. Traditional pharmacy curricula in China, however, focus predominantly on human health, with insufficient emphasis on veterinary and environmental perspectives. To address this gap, the present study focused on the student perspective, investigated Chinese pharmacy students' knowledge, attitudes, and practices (KAP) regarding environmental risks posed by PECs and their source control from a One Health perspective. The findings of this study are expected to provide valuable insights for better preparing qualified professionals for the effective source control of environmental risks posed by PECs in the future.

## 2 Methods

### 2.1 Research design

This cross-sectional observational survey was conducted using a self-designed online questionnaire via convenience sampling in November 2024. All the pharmacy students enrolled at Wuhan University of Science and Technology, a Chinese university offering undergraduate and master's degree pharmacy programs, were included in the study.

### 2.2 Survey instrument

Since no existing tools were available for assessing KAP related to environmental risks of PECs and their source control from a One Health perspective, a self-administered questionnaire was developed for this study. The questionnaire design was based on relevant literature regarding the One Health principle (10–14), as well as environmental risks posed by PECs and their source control (7–9, 11, 16). Three independent experts with expertise in the relevant fields evaluated the content validity, relevance, conciseness, and clarity of the questionnaire items. Additionally, a pilot test of the initial questionnaire was conducted among a convenience sample consisting of 10 second-year pharmacy undergraduates and 10 pharmacy graduate students. The Cronbach's α coefficient was 0.81, and the Kaiser-Meyer-Olkin (KMO) measure was 0.725. After minor revisions, the final questionnaire was distributed to all eligible pharmacy students, and data from the pilot test were excluded from the final analysis.

The final questionnaire consisted of 35 items, divided into four sections: (A) Demographic characteristics, including gender, age, degree program (undergraduate or master's), and academic year of study (Q1–Q4); (B) Knowledge assessment, comprising three five-point Likert scale items and 10 multiple-choice questions. Three Likert scale items (Q5–Q7) were used to evaluate participants' self-perceived understanding of One Health, environmental risks of PECs, and their source control, respectively. An additional 10 multiple-choice questions (Q8–Q17) were applied to objectively assess their actual knowledge levels. Specifically, the objective knowledge assessment included the following aspects: the concept (Q8) and core components (Q9) of One Health, the shared use of pharmaceuticals for treating human and animal diseases (Q10), the characteristics of PECs (Q11), the risks posed by PECs to exposed animals and plants (Q12), as well as environmental microbial communities (Q13), the typical concentration range of PECs in aquatic environment (Q14), the routes of pharmaceuticals to reach the environment (Q15), PECs included in the national list of priority ECs (Q16) (23), and a comparison in the half-lives of two common antibiotic classes, cephalosporins and fluoroquinolones, when present as ECs in the environment (Q17). For all multiple–choice questions with multiple correct answers, a response was scored 1 point only if all correct options were selected and no incorrect options were chosen. Incomplete answers, defined as responses that failed to select all correct options, as well as incorrect answers, were assigned a score of 0. The maximum score for the objective assessment of actual knowledge was 10 points; (C) Current concerns and practices, including five items assessing the students' current concerns about pharmaceutical residues in food products of plant/animal origin (Q18), environmental considerations during pharmaceutical consumption and use (Q19), practices related to drug storage (Q20) and disposal (Q21), as well as information sources on environmental risks of PECs (Q22); (D) Perceptions and attitudes, including 13 questions designed to investigate attitudes toward environmental risks of PECs and their source control from a One Health perspective (Q23–Q35), answered in a five-point Likert-scale format (1, strongly agree; 2, agree; 3, neutral; 4, disagree; and 5, strongly disagree).

The final questionnaire and study protocol were approved by the Ethics Committee of the School of Medicine, Wuhan University of Science and Technology.

### 2.3 Data collection

Links to the online questionnaire were distributed via class WeChat groups. Study objectives were explained in an introductory letter on the first page of the questionnaire, and digital informed consent was obtained from each participant prior to survey completion. The estimated time to complete the questionnaire was approximately 20 min. Participation was voluntary, and respondents remained anonymous throughout the study. Only fully completed questionnaires were included in subsequent analyses.

### 2.4 Statistical analysis

All the collected data were entered into SPSS 27.0 software (IBM Corp., USA) for statistical analysis. Qualitative data were presented as numbers and percentages with 95% confidence intervals (CI), and quantitative data were expressed as mean ± standard deviation (SD). Categorical variables were analyzed using the chi-square test or Fisher's exact test, as appropriate. Differences between two independent groups were compared using the independent samples *t*-test. One-way analysis of variance (ANOVA) followed by Tukey's *post-hoc* test was used for multiple group comparisons. Pearson correlation analysis was employed to explore the potential associations between the KAP dimensions related to environmental risks of PECs and their source control from the One Health perspective. Statistical significance was set at *p* < 0.05 or *p* < 0.01.

## 3 Results

### 3.1 Demographic characteristics of the respondents

A total of 287 pharmacy students enrolled in the studied university were invited to participate in the survey, yielding 206 valid questionnaires with an overall effective response rate of 71.8%. A summary of the demographic characteristics of the study participants is presented in Table 1. Among the 206 respondents, the majority were female (*n* = 148, 71.8%), and 57 were male (28.2%). The age distribution of the responding pharmacy students was approximately uniform around 20 years old. Regarding academic progression, 28.2, 19.9, 22.3, and 12.1% were in their first, second, third, and fourth years of the undergraduate program, respectively, with the remaining 17.5% being postgraduate students.

**Table 1:** Respondents' general demographic characteristics and knowledge levels regarding environmental risks of PECs and their source control from the perspective of One Health (*n* = 206).

Variables	Number (%; 95% CI)	Self-assessment for understanding						Objectively measured knowledge scores	
		One Health		Environmental risks of PECs		Source control for PECs-related environmental risks			
		Mean ±SD	*p*-Value	Mean ±SD	*p*-Value	Mean ±SD	*p*-Value	Mean ±SD	*p*-Value
Total		2.25 ± 0.99		2.48 ± 0.96		2.24 ± 0.93		2.48 ± 1.63	
Gender									
Male	58 (28.2%, 22.2–24.8)	2.17 ± 1.06	0.443	2.43 ± 1.03	0.575	2.24 ± 1.05	0.990	2.53 ± 1.48	0.768
Female	148 (71.8%, 65.2–77.8)	2.29 ± 0.96		2.51 ± 0.92		2.24 ± 0.89		2.46 ± 1.70	
Age									
≤ 20 years	99 (48.1%, 41.1–55.1)	2.44 ± 0.99	0.009^**^	2.55 ± 0.94	0.422	2.38 ± 0.96	0.036^*^	2.47 ± 1.58	0.961
>20 years	107 (51.9%, 44.9–58.9)	2.08 ± 0.96		2.44 ± 0.95		2.11 ± 0.89		2.49 ± 1.69	
Academic year of study									
1st year	58 (28.2%, 22.2–34.7)	2.59 ± 0.99	0.012^*^	2.52 ± 0.88	0.911	2.50 ± 0.96	0.046^*^	2.78 ± 1.56	0.058
2nd year	41 (19.9%, 14.7–25.8)	2.24 ± 0.97		2.59 ± 1.02		2.22 ± 0.94		2.05 ± 1.53	
3rd year	46 (22.3%, 16.8–28.5)	2.26 ± 0.95		2.46 ± 0.78		2.00 ± 0.67		2.11 ± 1.40	
4th year	25 (12.1%, 8.0–17.4)	2.04 ± 0.94		2.36 ± 1.08		2.00 ± 0.91		2.80 ± 1.56	
Graduate	36 (17.5%, 12.5–23.3)	1.89 ± 0.98		2.47 ± 1.08		2.33 ± 1.10		2.75 ± 2.03	

^*^p < 0.05; ^**^p < 0.01.

### 3.2 Knowledge levels regarding One Health, environmental risks of PECs, and their source control

As shown in Table 1, the average self-assessment scores of the 206 respondents on One Health, environmental risks of PECs, and their source control ranged from 2.24 ± 0.93 to 2.48 ± 0.96 out of a total of 5. However, the actual measured objective assessment scores were only 2.48 ± 1.63 out of 10, indicating extremely poor actual knowledge levels. When stratified by the students' demographic characteristics, group comparisons revealed significant differences in self-perceived knowledge scores by age and academic progression (*p* < 0.05; *p* < 0.01). First-year pharmacy undergraduates and students aged 20 years or younger exhibited greater confidence in their understanding of One Health and the source control of PECs-related environmental risks. However, no significant differences were observed in the objectively measured knowledge scores among the respondents (*p* > 0.05).

As shown in Table 2, among the 206 respondents, only 6.3% (95% CI: 3.0–9.6) answered correctly regarding the concept of One Health; 22.8% (95% CI: 17.1–28.5) fully and correctly identified the core components of One Health; and approximately one-third of the respondents (27.7%, 95% CI: 21.6–33.8) recognized that a considerable number of pharmaceuticals are simultaneously used for treating human and animal diseases. Regarding PECs, only 9.2% (95% CI: 5.3–13.1) and 10.2% (95% CI: 6.1–14.3) correctly identified the characteristics and environmental routes of PECs, respectively. The typical main concentration range (ng/L to μg/L) (7–9) of PECs occurred in aquatic environment was chosen by only a certain proportion (20.4%, 95% CI: 14.9–25.9) of students, but was wrongly underestimated by 47.6%, and overestimated by the remaining 32.0%. In addition, 35.4% (95% CI: 28.9–41.9) to 45.6% (95% CI: 38.8–52.4) of the respondents recognized the risks of PECs to exposed animals and plants, as well as environmental microbial communities. Moreover, 40.8% (95% CI: 34.1–47.5) of the respondents were aware that antibiotics have been included in the national list of priority ECs (23), and 29.6% (95% CI: 23.4–35.8) knew that cephalosporins have significantly shorter half-lives in the environment than fluoroquinolones (24).

**Table 2:** Accuracy of each question item in objectively measuring actual knowledge levels among pharmacy students who participated in the study (*n* = 206).

Question items	Number of respondents providing the correct answer	% of respondents providing the correct answer	95% CI
Q8: concept of One Health	13	6.3%	3.0–9.6
Q9: core components of One Health	47	22.8%	17.1–28.5
Q10: shared use of a considerable number of pharmaceuticals in both human and veterinary medicine	57	27.7%	21.6–33.8
Q11: characteristics of PECs	19	9.2%	5.3–13.1
Q12: risks of PECs in exposed animals and plants	73	35.4%	28.9–41.9
Q13: risks of PECs in environmental microbial communities	94	45.6%	38.8–52.4
Q14: typical concentration range of PECs in the aquatic environment	42	20.4%	14.9–25.9
Q15: routes of pharmaceuticals to reach the environment	21	10.2%	6.1–14.3
Q16: PECs included in the national list of priority ECs	84	40.8%	34.1–47.5
Q17: comparison of half-lives between two common classes of antibiotics, cephalosporins and fluoroquinolones, as ECs in the environment	61	29.6%	23.4–35.8

### 3.3 Current concerns and practices regarding environmental risks of PECs and their source control

**Figure 1:**
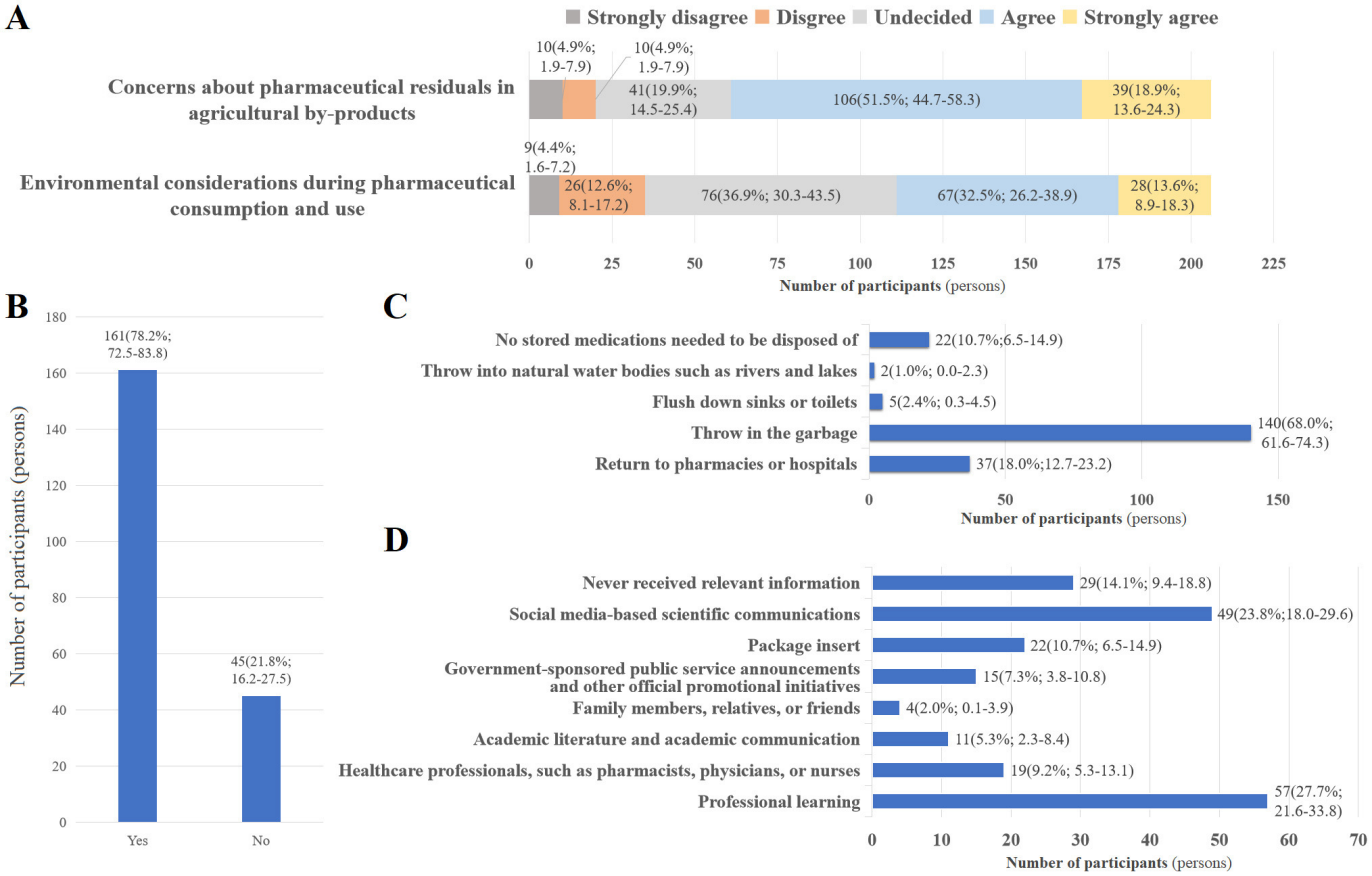
Current concerns and practices of pharmacy students regarding environmental risks of PECs and their source control. **(A)** Students' current concerns about pharmaceutical residues in food products of plant/animal origin, and environmental considerations during pharmaceutical use and consumption. **(B)** Responses to whether students store medications. **(C)** Graph showing preferred disposal methods for unused medications. **(D)** Information sources on environmental risks of PECs and their source control. Data were shown as numbers (%; 95% CI).

Subsequently, the responding students were surveyed to evaluate their current concerns and practices related to environmental risks of PECs and their source control. Firstly, we focused on the respondents' current concerns and considerations regarding two key issues in the field of environmental risks associated with PECs under the One Health framework, pharmaceutical residues in food and green alternatives to traditional chemical pharmaceuticals (10, 11, 25, 26). As shown in Figure 1A, more than 70% of the respondents agreed or strongly agreed that they were highly concerned about the presence of pharmaceuticals, such as antibiotics and hormones, in food products of plant/animal origin. However, less than half (46.1%) agreed or strongly agreed that they are currently worried about the potential environmental risks of pharmaceuticals, and try to choose environmentally friendly drugs as much as possible during consumption and use, while a considerable proportion of students (36.9%, 95% CI: 30.3–43.5) remained undecided.

Improper disposal of stored pharmaceuticals is widely recognized as an important source of PECs entering the environment, and is particularly regarded as a primary target for the source control of PECs-related environmental risks (16, 17, 27). Therefore, we evaluated the respondents' practices related to the storage and disposal of medications. As shown in Figure 1B, 78.2% (95% CI: 72.5–83.8) of the respondents reported that they stored medications for future use for “just in case” future use. A majority of the respondents (68.0%, 95% CI: 61.6–74.3) disposed of unwanted medications by discarding them in the garbage, while only 18.0% (95% CI: 12.7–23.2) opted to return such medications to pharmacies or hospitals (Figure 1C).

Regarding information sources on environmental risks of PECs and their source control (Figure 1D), the survey results showed that 27.7% (95% CI: 21.6–33.8) and 23.8% (95% CI: 18.0–29.6) of the respondents obtained relevant information via professional learning and social media-based scientific communications, respectively, which might act as the two primary information sources. However, 14.1% (95% CI: 9.4–18.8) of the respondents stated that they had never received such information.

Group comparative analyses revealed that only responses to two specific questions, pertaining to environmental considerations during pharmaceutical consumption/use and drug take-back practices, were significantly associated with age and academic advancement. Specifically, the number of students aged ≤ 20 years who reported agreeing or strongly agreeing that they take environmental factors into account when selecting and using pharmaceutical products was 53, significantly higher than the 42 students aged >20 years (*p* = 0.040). Regarding drug take-back behaviors, 27 younger students ( ≤ 20 years) indicated that they regularly return unused medications to hospitals or pharmacies, significantly more than the 10 older students (>20 years; *p* = 0.001). Additionally, stratified analysis by academic year showed that drug take-back practices were reported by 23 first-year undergraduates, four second-year undergraduates, four third-year undergraduates, two fourth-year undergraduates, and four postgraduate students, with a statistically significant difference observed across groups (*p* = 0.001).

Furthermore, we compared the knowledge scores of respondents with and without environmental considerations during pharmaceutical consumption/use. As shown in Table 3, students who incorporated environmental considerations into pharmaceutical use obtained significantly higher self-perceived knowledge scores regarding One Health and the source control of PECs-related environmental risks than their counterparts without such considerations (*p* < 0.01). Moreover, both the self-perceived and objectively measured knowledge scores of students who regularly returned unused medications to pharmacies or hospitals were markedly higher than those of students who opted for other drug disposal methods (Table 3, *p* < 0.05; *p* < 0.01). These data suggest that there may be significant associations between sustainable behaviors pertaining to medication use and the knowledge level regarding environmental risks of PECs and their source control from a One Health perspective among Chinese pharmacy students.

**Table 3:** Association between practices and knowledge levels regarding environmental risks of PECs and their source control among Chinese pharmacy student respondents (*n* = 206).

Practice	Self-assessment for understanding						Objectively measured knowledge scores	
	One Health		Environmental risks of PECs		Source control for PECs-related environmental risks			
	Mean ±SD	*p*-Value	Mean ±SD	*p*-Value	Mean ±SD	*p*-Value	Mean ±SD	*p*-Value
Respondent agreeing/strongly agreeing with their current environmental considerations during pharmaceutical consumption/use (*n* = 95)	2.47 ± 0.98	0.004^**^	2.62 ± 0.88	0.066	2.45 ± 0.87	0.003^**^	2.57 ± 1.58	0.477
Respondents without environmental considerations during pharmaceutical consumption/use (*n* = 111)	2.07 ± 0.97		2.38 ± 0.99		2.06 ± 0.95		2.41 ± 1.68	
Respondents returning unwanted medications to pharmacies or hospitals (*n* = 37)	2.76 ± 1.16	0.000^**^	2.81 ± 1.02	0.037^*^	2.84 ± 1.17	0.000^**^	3.05 ± 1.43	0.011^*^
Respondents engaging other drug disposal efforts (*n* = 169)	2.15 ± 0.92		2.42 ± 0.92		2.11 ± 0.82		2.36 ± 1.65	

^*^p < 0.05; ^**^p < 0.01.

### 3.4 Attitudes toward environmental risks of PECs and their source control from the perspective of One Health

Table 4 demonstrated that, in general, pharmacy students held positive perceptions toward environmental risks of PECs and their source control from the One Health perspective. A substantial majority of respondents (79.6%) agreed or strongly agreed that pharmacy professional practice ought to be sufficiently aligned with the One Health concept. The adverse effects of PECs on the vertices of the “One Health triangle” (humans, animals, and the environment) were recognized by 83.0%−87.4% of the participating students. Regarding the reverse-scored item “Pharmacy professionals' participation in environmental risk control of PECs and is not essential, as the responsibility for this task lies mainly with environmental experts and regulators,” only 38 (18.4%) pharmacy students agreed or strongly agreed, while 66.5% (*n* = 137) disagreed, indicating that most pharmacy students recognize that environmental risk control of PECs is not solely the responsibility of environmental experts and regulators, but also that of pharmacy professionals. Encouragingly, an overwhelming majority of students (84.5%) stated that they would strongly support and actively participate in the implementation of source-control interventions aimed at reducing environmental emissions of PECs. In terms of specific source-control measures for PECs-related environmental risks, 86.4% of respondents agreed or strongly agreed with the incorporation of environmental sustainability considerations into the evaluation framework for rational drug use (28, 29); 122 students (59.2%) supported the mandatory implementation of a drug take-back system for the rational disposal of unused and expired medicines, which serves as a critical step in mitigating environmental risks of PECs (27); and 85.9% of Chinese pharmacy students acknowledged the necessity of conducting an environmental risk assessment prior to the marketing approval of a new drug, a practice already implemented in the European Union and the United States (30). Additionally, most respondents endorsed the integration of One Health principle, encompassing human, veterinary, and environmental perspectives (20), into the pharmaceutical professional knowledge system (*n* = 177, 85.9%), stated that acquiring knowledge about environmental risks of PECs and their source control is highly beneficial to their future career development as pharmacy professionals (*n* = 181, 87.9%), and 81.1% (*n* = 167) expressed the intention to deepen their understanding of this relevant knowledge.

**Table 4:** Attitudes of pharmacy students toward environmental risks of PECs and their source control from the perspective of One Health (*n* = 206).

Survey question/statement	Responses, number (%; 95% CI)				
	Strongly disagree	Disagree	Undecided	Agree	Strongly agree
Q23: the practice of the pharmacy profession need to be sufficiently aligned with the One Health concept.	6 (2.9%; 0.6–5.2)	4 (1.9%; 0.1–3.8)	32 (15.5%; 10.6–20.5)	100 (48.5%; 41.7–55.4)	64 (31.1%; 24.8–37.4)
Q24: PECs exert severe adverse impacts on environmental health, thereby warranting special attention.	3 (1.5%; 0.0–3.1)	2 (1.0%; 0.0–2.3)	27 (13.1%; 8.5–17.7)	103 (50.0%; 43.2–56.8)	71 (34.5%; 28.0–41.0)
Q25: PECs exert severe adverse impacts on animals, thereby warranting special attention.	5 (2.4%; 0.3–4.5)	1 (0.5%; 0.0–1.5)	28 (13.6%; 9.0–18.3)	100 (48.5%; 41.7–55.4)	71 (34.5%; 28.0–41.0)
Q26: PECs exert severe adverse impacts on human health, thereby warranting special attention	3 (1.5%; 0.0–3.1)	1 (0.5%; 0.0–1.5)	22 (10.7%; 6.5–14.9)	103 (50.0%; 43.2–56.8)	77 (37.4%; 30.8–44.0)
Q27: occurrence of PECs in the environment can lead to the emergence of drug-resistant strains and the spread of drug-resistant genes.	5 (2.4%; 0.3–4.5)	1 (0.5%; 0.0–1.5)	24 (11.7%; 7.3–16.1)	114 (55.3%; 48.6–62.1)	62 (30.1%; 23.9–36.3)
Q28: pharmacy professionals' participation in environmental risk control of PECs is not essential, as the responsibility for this task lies mainly with environmental experts and regulators.	52 (25.2%; 19.3–31.2)	85 (41.3%; 34.6–48.0)	31 (15.1%; 10.2–20.0)	23 (11.2%; 6.9–15.5)	15 (7.3%; 3.8–10.8)
Q29: I would strongly support and actively participate in the implementation of source-control interventions aimed at reducing environmental emissions of PECs.	2 (1.0%; 0.0–2.3)	5 (2.4%; 0.3–4.5)	25 (12.1%; 7.7–16.6)	109 (52.9%; 46.1–59.7)	65 (31.6%; 25.3–37.9)
Q30: considerations related to environmental sustainability should be incorporated into the evaluation framework for rational drug use.	2 (1.0%; 0.0–2.3)	5 (2.4%; 0.3–4.5)	21 (10.2%; 6.1–14.3)	111 (52.9%; 47.1–60.7)	67 (32.5%; 26.2–38.9)
Q31: drug take-back system for rational disposal of unused and expired medicines should be mandatory, as it constitutes a critical step in mitigating environmental risks of PECs.	3 (1.5%; 0.0–3.1)	13 (6.3%; 3.0–9.6)	68 (33.0%; 26.6–39.4)	78 (37.9%; 31.3–44.5)	44 (21.4%; 15.8–27.0)
Q32: an environmental risk assessment should be performed before a new drug is approved for marketing to ensure its environmental friendliness.	3 (1.5%; 0.0–3.1)	1 (0.5%; 0.0–1.5)	19 (9.2%; 5.3–13.1)	116 (56.3%; 49.6–63.0)	67 (32.5%; 26.2–38.9)
Q33: the One Health principle, encompassing human, veterinary and environmental perspectives, constitutes a vital component of the pharmaceutical professional knowledge system.	3 (1.5%; 0–3.1)	3 (1.5%; 0–3.1)	23 (11.2%; 6.9–15.5)	118 (57.3%; 50.5–64.0)	59 (28.6%; 22.5–34.8)
Q34: acquiring knowledge about environmental risks of PECs and their source control is highly beneficial to my future career development as a pharmacy professional.	4 (1.9%; 0.1–3.8)	2 (1.0%; 0.0–2.3)	19 (9.2%; 5.3–13.1)	125 (60.7%; 54.0–67.3)	56 (27.2%; 21.1–33.3)
Q35: I intend to deepen my knowledge about environmental risks of PECs and their source control from the perspective of One Health.	4 (1.9%; 0.1–3.8)	1 (0.5%; 0.0–1.5)	34 (16.5%; 11.5–21.5)	114 (55.3%; 48.6–62.1)	53 (25.7%; 19.8–31.7)

Significant gender-based differences were observed in responses to Q28, Q29, Q30, and Q32, all of which were assessed using a five-point Likert scale. More females opposed to the reverse-scored item “Pharmacy professionals' participation in environmental risk control of PECs and is not essential, as the responsibility for this task lies mainly with environmental experts and regulators” (Q28, female 3.84 ± 1.11, male 3.19 ± 1.24, *p* = 0.000). Additionally, females exhibited greater positivity toward supporting and actively participating in the implementation of source-control interventions aimed at reducing environmental emissions of PECs (Q29: 4.20 ± 0.70 vs. 3.91 ± 0.86 for males; *p* = 0.019), held more favorable attitudes toward specific source-control measures, including the integration of environmental sustainability considerations into the evaluation framework for rational drug use (Q30: 4.20 ± 0.74 vs. 3.93 ± 0.86 for males; *p* = 0.012) and the conduct of environmental risk assessments prior to the approval of new drugs for marketing (Q32: 4.24 ± 0.66 vs. 4.02 ± 0.89 for males; *p* = 0.047). These data indicated that females held more positive attitudes toward the source control for PECs-related environmental risks. Responses to all other attitude-related questions were not significantly associated with demographic factors.

As presented in Table 5, Pearson's correlation analysis of the variables related to the KAP dimensions regarding environmental risks of PECs and their source control from a One Health perspective revealed that the attitude scores were positively correlated with self-perceived knowledge about One Health (*r* = 0.187, *p* < 0.01), self-perceived knowledge about source control of PECs-related environmental risks (*r* = 0.148, *p* < 0.05), or environmental considerations in pharmaceutical consumption/use (*r*= 0.248, *p* < 0.01).

**Table 5:** Pearson's correlation coefficients between KAP dimensions of environmental risks of PECs and their source control from the perspective of One Health (*n* = 206).

Variables	1	2	3	4	5	6	7
**1** Self-perceived knowledge about One Health	1						
**2** Self-perceived knowledge about environmental risks of PECs	0.605^**^	1					
**3** Self-perceived knowledge about source control for PECs-related environmental risks	0.637^**^	0.718^**^	1				
**4** Objectively measured knowledge	0.227^**^	0.270^**^	0.311^**^	1			
**5** Environmental considerations during pharmaceutical consumption/use	0.202^**^	0.128	0.209^**^	0.050	1		
**6** Drug take-back practice	0.236^**^	0.159^*^	0.300^**^	0.165^*^	0.277^**^	1	
**7** Attitudes toward environmental risks of PECs and their source control from the perspective of One Health	0.187^**^	0.085	0.148^*^	−0.050	0.248^**^	−0.027	1

^*^p < 0.05; ^**^p < 0.01.

## 4 Discussion

With the growing concerns about environmental risks of PECs and the global advancement of sustainability within higher education (31), embedding sustainability into pharmacy education has recently been identified as critical to preparing future pharmacists and other pharmacy professionals to deliver optimized pharmaceutical services balancing high-quality pharmacotherapy with environmental safety (2, 21, 32, 33). The components of environmental sustainability in pharmacy practice are multifaceted, with previous studies mainly emphasizing climate change associated with pharmaceutical manufacturing and the social accountability of pharmacy schools, as reviewed by Urslak et al. (21). The present study focuses on environmental risks of PECs, an emerging and increasingly prominent topic within the broad framework of environmental sustainability in pharmacy practice. From an expert perspective, future pharmacy professionals are expected to contribute to the source control of PECs-induced environmental risks by developing eco-friendly pharmaceuticals, advancing green chemistry in pharmaceutical manufacturing processes, monitoring and minimizing pharmaceutical emissions to the environment, implementing eco-directed sustainable prescribing, scientifically managing unused medications, promoting proper disposal and take-back programs, and disseminating information and knowledge regarding the environmental risks of PECs and their mitigation measures (14). Therefore, relevant competencies should be fostered through professional education. However, current pharmacy professional training systems worldwide generally overlook environmental aspects pertaining to pharmaceuticals. As recipients of education, pharmacy students are both beneficiaries of and key stakeholders in professional training; thus, student perspectives should also be considered when integrating sustainability into pharmacy education. The present study assessed the KAP levels of Chinese pharmacy students regarding environmental risks of PECs and their source control from a One Health perspective. Results indicated that most Chinese pharmacy students held positive attitudes and strong intentions toward the One Health-based source control for environmental risks of PECs, but they lacked sufficient knowledge and demonstrated inadequate practices. This finding is encouraging, as translating such positive attitudes and intentions into behaviors may facilitate active participation of future pharmacy professionals in addressing environmental risks posed by PECs. However, relevant knowledge should be imparted and reinforced during pharmacy education.

Surveys conducted among pharmacy students in Spain (2, 32) reported that approximately 75%−90% of responding students stated that they were unaware of concepts related to ECs, One Health, or “Environmental Risk Assessments,” the latter of which have been mandatory for human and veterinary medicines in Spain under the European Medicines Agency regulations since 2005. Another study conducted in Ethiopia found that nearly 80% of pharmacy students had not read any reports or articles about pharmaceutical environmental pollution (22). Consistent with these prior self-reported data, the present study revealed that the average self-perceived knowledge scores for One Health, environmental risks of PECs, and their source control were only 2.25, 2.48, and 2.24 out of a total of 5, respectively, suggesting that Chinese pharmacy students lack self-confidence in understanding basic concepts in the fields of PECs-related environmental risks and their source control from the perspective of One Health. These data collectively verify that sustainability is currently rarely integrated into global pharmacy teaching in both HICs and LMICs, and promoting professional education on environmental risks of PECs may be a critical topic for pharmacy schools around the world to address the environmental challenges posed by pharmaceuticals. Given that the use of self-reported measures may introduce response bias, the present study employed an addition 10 close-ended questions to objectively measure the actual understanding of Chinese pharmacy students, and the results showed that the average actual knowledge score was merely 2.48 out of a maximum of 10 points, which was lower than their self-perceived scores. Interestingly, group comparisons indicated that first-year and younger undergraduates appeared more confident in their own understanding of One Health, environmental risks of PECs, and their source control; however, their actual knowledge scores were not significantly higher than their senior counterparts. Such “overconfidence” (34) might be attributed to freshmen's inadequate grasp of professional knowledge, which prevents them from fully comprehending the complexity of these topics. Therefore, environmental sustainability related to environmental risks of PECs and their source control should be integrated not only into core curricula during professional learning stage, but also embedded into professional awareness-building education in the first year of undergraduate studies, thereby motivating freshmen to engage in further learning in this area. In the objective knowledge assessment, only 6.3%−10.2% of students correctly answered questions concerning the One Health concept, characteristics of PECs, and pathways through which pharmaceuticals enter the environment, which indicated insufficient formal educational training on these topics. Additionally, we found that 47.6% of respondents underestimated the typical concentration range of PECs in the aquatic environment, indicating that a considerable proportion of pharmacy students underestimate the environmental risks of PECs.

Under the One Health framework, residues of antibiotics, growth-promoting agents, and other veterinary pharmaceuticals in food-producing animals and plants have garnered widespread societal attention, primarily due to their associated human health risks including antibiotic resistance, hormonal disruptions, and allergic reactions (10, 25, 35). Notably, this societal concern was also highlighted by the majority of pharmacy students participating in the present study. By contrast, fewer than half of the respondents reported incorporating environmental considerations into their pharmaceutical consumption and use. In recent years, eco-directed sustainable prescribing (EDSP), which advocates for the prescription of pharmaceuticals at low doses and with environmentally benign properties, has been increasingly established as an optimized decision-support tool in clinical prescribing practice (28, 29). EDSP aims to integrate environmental considerations into drug prescribing, thereby mitigating the environmental discharge of PECs derived from human and animal excretion at the source of drug use (28, 29). The insufficient integration of environmental considerations into pharmaceutical consumption and use among pharmacy students, as identified in the present survey, may impede their future capacity to engage as pharmacists and other pharmacy professionals in promoting sustainable pharmaceutical practices. Furthermore, the present study found that 78.2% of Chinese pharmacy students self-reported storing unused medications. This proportion is notably higher than that reported among pharmacy students in Saudi Arabia (37.6%) (36) and Ethiopia (64%) (22). A plausible explanation for this discrepancy is that the survey period of this study coincided with a large-scale influenza outbreak in China, prompting students to stockpile medications for preventive and therapeutic use. If these stored medications are improperly disposed of upon expiration or when no longer needed, the environmental load of PECs will be further exacerbated (16, 17, 27). To date, drug take-back systems which enable the public to return unused pharmaceuticals to certified collection points such as pharmacies and hospitals have been widely recognized as safe, eco-friendly, and legally compliant solutions for medication waste disposal (16, 17, 27). However, fewer than one-fifth of the students in our study reported returning unused medications to pharmacies or hospitals. Although this proportion is higher than those reported in Saudi Arabia (8.1%) (36) and Ethiopia (6.1%) (22), there remains substantial room for improvement in the drug take-back practices of Chinese pharmacy students, given their future pivotal roles as pharmacists in advocating for and facilitating drug take-back programs.

In the attitude dimension, consistent with previous findings from Spain (2, 32) and Ethiopia (22), the present study revealed that Chinese pharmacy students also hold highly positive attitudes toward the source control for environmental risks posed by PECs from a One Health perspective and toward acquiring related knowledge, with an average score of 4.05 ± 0.61 across all 13 attitude-related items assessed using a 5-point Likert scale. Most students endorsed the integration of the One Health principle and knowledge related to environmental risks of PECs into the pharmaceutical professional curriculum, as a means to enhance their future career development as pharmacy practitioners. Additionally, professional learning was identified as the most common information source for understanding environmental risks of PECs and their source control. In fact, the university involved in this study had not systematically and intentionally integrated environmental sustainability-related content into its pharmacy curriculum prior to the survey. In recent years, several pioneering institutions have begun attempting to equip pharmacy students and professionals with sustainability knowledge (2, 37, 38). For example, the Department of Pharmacy at the University of Huddersfield in the United Kingdom is integrating environmental sustainability principles, including the roles of pharmacists and pharmacy teams in addressing climate change and reducing carbon emissions, into its Master of Pharmacy curriculum to “green” the program (37). The University of Helsinki in Finland launched a holistic initiative known as the Generation Green task force in 2015, aiming to incorporate sustainability issues related to drug production and use into pharmacy education. Subsequently, this university established the world's first Professorship in Sustainable Pharmacy in 2023 (2, 38). According to the experience of the University of Helsinki, the students' attitudes, needs and interests should not be overlooked during sustainability-integrated curriculum reform (38). Given the prevalent issue of “curricular overload” in pharmacy programs globally, Canadian pharmacy educators (19) have advised that sustainability-related content should not be added as standalone modules, and can be thoughtfully integrated into existing components of pharmacy education. While efforts to embed sustainability into pharmacy curricula are still in their infancy, the positive attitudes and strong willingness of Chinese pharmacy students to acquire knowledge about environmental risks of PECs and their source control from a One Health perspective, identified in the present study, is encouraging, as these will help prepare students to fulfill their future roles as key stakeholders in the source control for environmental risks of PECs arising from both human and veterinary pharmacotherapies. Moreover, compared with previous initiatives in attempt to introduce a systematic sustainability framework (including climate change, carbon emissions, etc.) in pharmacy education (2, 37, 38), our findings suggested that, from the students' standpoint, the environmental risks of PECs and their source control from a One Health perspective could serve as an engaging and concretized topic of sustainability-related issues closely aligned with the pharmacy profession.

Correlation analysis among the KAP dimensions regarding environmental risks of PECs and their source control from a One Health perspective showed that knowledge level and confidence in their understanding might exert a positive influence on pharmacy students' sustainable pharmaceutical practices and attitudes, which further underscores the critical role of enhancing knowledge and awareness of One Health, environmental risks of PECs, and their source control in preparing future pharmacy professionals to address environmental challenges posed by the development of pharmaceutical industry. Under the framework of One Health education (39), sustainable topics related to zoonotic disease management, responsible antimicrobial stewardship, the detection, assessment, understanding, and prevention of adverse effects of PECs could be incorporated into the core courses of pharmaceutical professional education (20). In doing so, students can comprehensively develop the professional skills, interprofessional knowledge, and systems-thinking required in sustainable pharmaceutical practices to address pressing and complex global health and environmental issues. The integration of One Health, environmental risks of PECs and their source control into the professional education would empower pharmacy professionals working at the intersection of human, animal, and environmental health to mitigate the adverse environmental impacts of pharmaceuticals through collaboration with other key stakeholders, including environmental regulators, healthcare institutions, and veterinarian workers (20, 39).

## 5 Limitations

This study provides initial insights into the environmental risks of pharmaceuticals and their source control under the One Health framework, from the perspective of pharmacy students. However, it has some limitations that should be considered when interpreting the findings. First, the study sample was relatively small, which was insufficient for checking structural validity and conducting Exploratory Factor Analysis (EFA) or Confirmatory Factor Analysis (CFA). Further studies with larger sample sizes are therefore required to provide evidence of construct validity (e.g., EFA/CFA), conduct separate reliability assessments for each KAP domain, and collect test-retest reliability data, thereby validating the psychometric robustness of the questionnaire. Moreover, the study population consisted of students whose curriculum largely lacked relevant knowledge on the topics investigated. In particular, the large proportion of first-year students with limited understanding of the subject may have introduced response bias. Further investigations should be conducted among professionals in service. In addition, the single-center design combined with WeChat-based voluntary recruitment may have led to selection bias and non-response bias, potentially restricting the external validity of the results. As convenience sampling was adopted in this study, no sample size calculation was performed. Correlations among the KAP dimensions were initially assessed using Pearson's correlation analysis, but the lack of adjustment for multiple comparisons and control for potential confounding factors may weaken the reliability of the reported associations. Future studies adopting multi–center designs, probability sampling, and adjusted statistical models are warranted to validate the findings and minimize methodological bias.

## 6 Conclusion

The present study focused on One Health, environmental risks of PECs, and their source control, prominent issues in the field of environmental sustainability, and highlighted the need to strengthen these sustainable topics in pharmacy education to equip future pharmacy professionals with the capacity to effectively address increasingly severe risks caused by PECs in the environment. The majority of responding Chinese pharmacy students had a positive perception toward environmental risks of PECs and their source control from a One Health perspective, but lacked relevant practice and knowledge. These findings indicate that future pharmacy professionals are inadequately prepared to fulfill their role in the source control of PECs-related environmental risks, thereby underscoring the urgent imperative to enhance educational and training initiatives centered on environmental sustainability with respect to environmental risks of PECs. In particular, knowledge level and confidence in one's understanding might exert a positive influence on pharmacy students' sustainable pharmaceutical practices and attitudes. This gap reinforces the importance of customizing the integration of sustainability content, related to One Health, environmental risks of PECs and their source control, into existing components of the professional curriculum to embed sustainability-related competencies in pharmacy education and translate students' positive attitudes into professional action. Encouragingly, the responding students expressed clear support and a strong desire for such integration into their professional training.

## Data Availability

The raw data supporting the conclusions of this article will be made available by the authors, without undue reservation.
